# Investigating the potential causal association between consumption of green tea and risk of lung cancer: a study utilizing Mendelian randomization

**DOI:** 10.3389/fnut.2024.1265878

**Published:** 2024-02-19

**Authors:** Jieming Lu, Ye Lin, Junfei Jiang, Lei Gao, Zhimin Shen, Changping Yang, Pinghua Lin, Mingqiang Kang

**Affiliations:** ^1^Department of Thoracic Surgery, Fujian Medical University Union Hospital, Fuzhou, China; ^2^Key Laboratory of Cardio-Thoracic Surgery, Fujian Medical University, Fuzhou, China; ^3^Key Laboratory of Gastrointestinal Cancer (Fujian Medical University), Ministry of Education, Fuzhou, China; ^4^Fujian Key Laboratory of Tumor Microbiology, Department of Medical Microbiology, Fujian Medical University, Fuzhou, China; ^5^Fuqing City Hospital Affiliated to Fujian Medical University, Fuzhou, China

**Keywords:** lung cancer, green tea, Mendelian randomization, polyphenols, epigallocatechin-3-gallate (EGCG), Genome-Wide Association Study (GWAS)

## Abstract

**Background:**

Lung cancer is the most common global cancer in terms of incidence and mortality. Its main driver is tobacco smoking. The identification of modifiable risk factors isa public health priority. Green tea consumption has been examined in epidemiological studies, with inconsistent findings. Thus, we aimed to apply Mendelian randomization to clarify any causal link between green tea consumption and the risk of lung cancer.

**Methods:**

We utilized a two-sample Mendelian randomization (MR) approach. Genetic variants served as instrumental variables. The goal was to explore a causal link between green tea consumption and different lung cancer types. Green tea consumption data was sourced from the UK Biobank dataset, and the genetic association data for various types of lung cancer were sourced from multiple databases. Our analysis included primary inverse-variance weighted (IVW) analyses and various sensitivity test.

**Results:**

No significant associations were found between green tea intake and any lung cancer subtypes, including non-small cell lung cancer (adenocarcinoma and squamous cell carcinoma) and small cell lung cancer. These findings were consistent when applying multiple Mendelian randomization methods.

**Conclusion:**

Green tea does not appear to offer protective benefits against lung cancer at a population level. However, lung cancer's complex etiology and green tea's potential health benefitssuggest more research is needed. Further studies should include diverse populations, improved exposure measurements and randomized controlled trials, are warranted.

## Introduction

Tea is one of the most widely consumed beverages in the world, known not just for its flavors but also for its potential health benefits. It is a rich source of polyphenols, specifically a catechin called epigallocatechin-3-gallate (EGCG) (1). Prior research has unveiled the antioxidant, anti-inflammatory (2), and anticarcinogenic properties of EGCG (3, 4), opening the possibility that regular consumption of tea, particularly green tea, might offer protective effects against various health conditions, including cancer.

Lung cancer, primarily driven by tobacco smoking, remains the most common cancer globally, both in terms of incidence and mortality (5). Despite the well-established role of smoking in lung cancer development (6, 7), other environmental factors (8, 9) and lifestyle factors (10, 11) are also important, and their influence on the causes of lung cancer is still an active area of research. Given the severity and prevalence of lung cancer, identifying additional modifiable risk factors is a high priority in public health. Among these, diet has received increasing attention (12, 13), making the exploration of dietary elements like green tea crucial.

Epidemiologically, the relationship between green tea consumption and lung cancer risk has been extensively studied, but the findings have been inconsistent. Some observational studies and meta-analyses suggest a protective effect of green tea (14, 15), particularly among non-smokers (16), while others have found null associations (17, 18). These discrepancies can be attributed to several factors such as variations in tea consumption habits, differences in the preparation method and type of tea, and potential confounding by tobacco smoking and other lifestyle factors.

Mendelian randomization (MR), an epidemiological tool that leverages genetic variants as instrumental variables, offers a unique opportunity to mitigate such biases inherent to observational studies (19). In our studies, genetic variants strongly associated with an exposure, the consumption of green tea, are used to form an instrumental variable. Since these genetic variants are determined at conception, they are not prone to reverse causation, and they should be unrelated to the confounding factors that typically bias observational studies. By using genetically predicted exposures, MR studies can provide less confounded estimates of causal effects, thereby overcoming limitations of conventional epidemiological studies (20). Despite its potential, to our knowledge, the MR approach has not yet been applied to investigate the causal effect of green tea consumption on lung cancer risk.

Therefore, our findings aim to add a new perspective to the existing literature on the relationship between green tea consumption and lung cancer risk. By employing Mendelian randomization, this study seeks to clarify the potential causal links that have been previously obscured in observational studies. While our results may not directly influence the current understanding of lung cancer etiology, they provide a foundation for future research in this area, potentially guiding more targeted investigations into preventive strategies.

## Methods

### Study design

**Figure 1:**
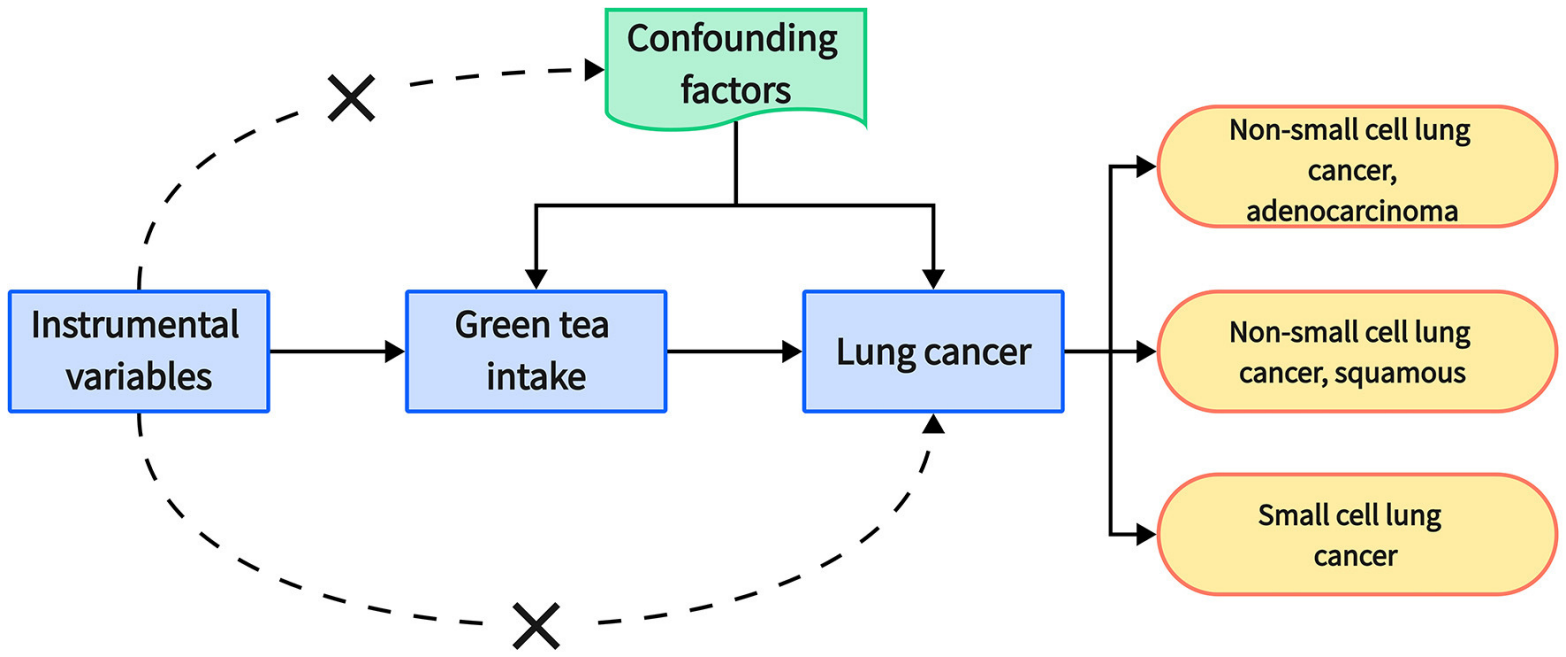
Schematic representation of Mendelian randomization analysis framework. It illustrates the sequence of analysis: starting with instrumental variables that are genetic variants associated with green tea intake, the pathway leads to lung cancer, highlighting subtypes such as adenocarcinoma and squamous non-small cell lung cancer, and small cell lung cancer. Confounding factors are denoted as being separate from the causal pathway. This visual underscores the exclusion of confounding variables in the Mendelian randomization approach and delineates the hypothesized influence of green tea consumption on lung cancer subtypes.

This study employed a two-sample Mendelian randomization (MR) approach to investigate the potential causal relationship between green tea consumption and the risk of distinct types of lung cancer, namely, non-small cell lung cancer (NSCLC) (with further division into adenocarcinoma and squamous cell carcinoma subtypes) and small cell lung cancer (SCLC) (Figure 1).

**Figure 2:**
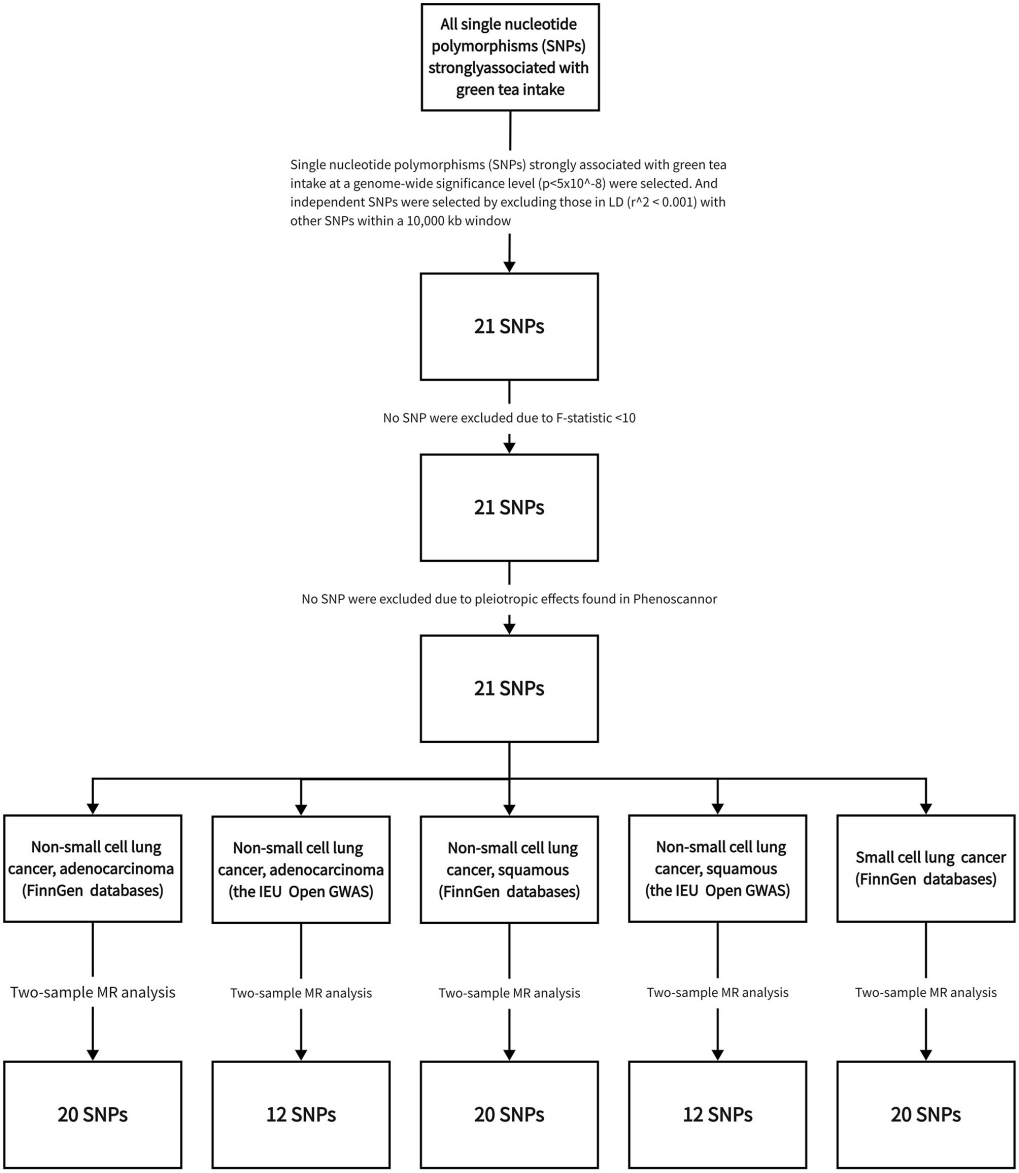
Flowchart illustrating the methodology of Mendelian randomization analysis utilized in the current investigation.

The detailed characteristics of the data implemented in this study are comprehensively captured in Table 1. Meanwhile, the step-by-step procedure of the SNP selection process, integral to our analysis, along with the derived results are graphically represented in Figure 2.

**Table 1:** Characteristics of the data implemented in this study.

**Traits**	**Data sources**	**GWAS id**	**Cases**	**Control**	**Number of SNP**	**Ancestry**
**Exposure**						
Green tea intake	the UK Biobank dataset	ukb-b-4078	64,949	-	9,851,867	European
**Outcomes**						
Non-small cell lung cancer, adenocarcinoma	FinnGen databases	finn-b-C3_NSCLC_ADENO	571	218,221	16,380,466	European
Non-small cell lung cancer, adenocarcinoma	the IEU Open GWAS	ieu-a-965	3,442	14,894	8,881,354	European
Non-small cell lung cancer, squamous	FinnGen databases	finn-b-C3_NSCLC_SQUAM	365	218,427	16,380,466	European
Non-small cell lung cancer, squamous	the IEU Open GWAS	ieu-a-967	3,275	15,038	8,893,750	European
Small cell lung cancer	FinnGen databases	finn-b-C3_SCLC	179	218,613	16,380,466	European

### Genome-Wide Association Study procedure

To investigate the causal relationships between green tea consumption and lung cancer subtypes, we utilized a Genome-Wide Association Study (GWAS) approach. GWAS enables the comprehensive examination of common, low-penetrance genetic variants and their association with specific phenotypes, in this case, lung cancer and its subtypes (21).

In this study, we carried out a Genome-Wide Association Study (GWAS) to identify single nucleotide polymorphisms (SNPs) that are associated with green tea consumption and its potential effect on lung cancer risk. The GWAS was performed following a well-structured workflow, which is based on established guidelines and procedures. Samples were collected from volunteers participating in the UK Biobank, following informed consent and ethical approval. Genotyping was done using the Affymetrix Axiom UK BiLEVE array, and subsequent quality control measures were applied (22).

### Exposure data and instrumental variable selection

The exposure of interest in our study was green tea consumption. We sourced data from the UK Biobank dataset (Green tea intake Dataset: ukb-b-4078), which consists of 64,949 participants of European descent, both males and females, who reported their green tea intake.

From this dataset, we identified single nucleotide polymorphisms (SNPs) strongly associated with green tea intake at a genome-wide significance level (p < 5x10^∧^-8) in the genome-wide association study (GWAS). These SNPs were employed as instrumental variables (IVs) for green tea consumption, providing a means of approximating the randomized exposure necessary for our Mendelian randomization analysis.

To ensure the validity of our IVs and to avoid bias introduced by the co-inheritance of genetic variants, we conducted linkage disequilibrium (LD) pruning. We selected independent SNPs by excluding those in LD (r^∧^2 < 0.001) with other SNPs within a 10,000 kb window. This stringent LD threshold and window size ensured the selected SNPs were independent and reduced the likelihood of biased MR estimates due to correlated instruments.

We further refined the selection of our IVs through a series of steps. Firstly, we calculated the F-statistic for each SNP to measure the strength of the IVs. All SNPs yielded an F-statistic of > 10, indicating a low risk of weak instrument bias; thus, no SNP was excluded at this step.

Next, we scrutinized the selected SNPs using the PhenoScanner database to assess whether these SNPs were associated with established lung cancer risk factors (*P* < 5 × 10–8), such as age and smoking. SNPs associated with these confounders were also excluded to minimize confounding bias.

The resulting set of SNPs, which were independently and robustly associated with green tea intake and not with known confounders, were then employed as instrumental variables in our subsequent Mendelian randomization analyses.

### Outcome data

We sourced genetic association data for various types of lung cancer from multiple databases to evaluate the potential impact of green tea consumption on lung cancer risk.

The first dataset involved non-small cell lung cancer (NSCLC), specifically adenocarcinoma, from the FinnGen study (Dataset: finn-b-C3_NSCLC_ADENO). This dataset included 571 cases and 218,221 controls, all of European descent and comprising both males and females.

In addition, we used the lung adenocarcinoma dataset from the IEU Open GWAS database (Dataset: ieu-a-965), comprising a European population of both sexes with 3,442 cases and 14,894 controls.

We extracted data for NSCLC of the squamous cell subtype from the FinnGen study (Dataset: finn-b-C3_NSCLC_SQUAM). This dataset involved a European population of both sexes, with 365 cases and 218,427 controls.

Also, the squamous cell lung cancer dataset from the IEU Open GWAS database (Dataset: ieu-a-967) was used, including 3,275 cases and 15,038 controls from a European population of both sexes.

Lastly, we used the small cell lung cancer (SCLC) dataset from the FinnGen study (Dataset: finn-b-C3_SCLC). This encompassed a European population of both sexes, with 179 cases and 218,613 controls.

Each of these datasets provided SNP-wise association summary statistics from respective genome-wide association studies (GWAS). These data were used for the Mendelian randomization analysis to examine the putative causal relationships.

### Mendelian randomization analysis

We conducted a two-sample MR using several approaches. The main analysis was performed using the inverse variance-weighted (IVW) method. The MR-Egger regression, weighted median method, and weighted mode-based estimator were employed for sensitivity analyses. These methods provide different assumptions about the pleiotropy of the genetic instruments and are used to test the robustness of the findings.

The MR-Egger regression can provide a valid causal estimate even when all genetic variants are invalid instruments, as long as the instrument strength independent of direct effect (InSIDE) assumption holds. The weighted median method can provide a correct estimate if at least 50% of the weight in the analysis comes from valid instruments. The weighted mode-based estimator will give a consistent estimate if the largest number of similar individual-instrument causal effect estimates comes from valid instruments.

### Statistical analysis

#### Methodology overview: Mendelian randomization analysis

In this study, we employed a two-sample Mendelian Randomization (MR) approach to investigate the potential causal relationship between green tea consumption and the risk of developing various types of lung cancer.

#### Computational tools and software

The statistical analyses were performed using R software, version 4.2.1. For the MR analyses, we utilized two specific R packages: “MendelianRandomization” and “TwoSampleMR”.

#### Primary analysis approach: Inverse Variance-Weighted method

As part of our primary analysis, the Inverse Variance-Weighted (IVW) method was applied. This method aggregates the estimated effects of individual genetic variants, weighted by their precision, to provide an overall causal effect estimate.

#### Sensitivity analyses

MR-Egger Regression: We used this method as it allows for a valid causal inference even in the presence of pleiotropic effects, provided that the InSIDE (Instrument Strength Independent of Direct Effect) assumption is met.

Weighted Median Method: This method can yield a correct causal estimate when at least 50% of the weight in the analysis comes from valid instrumental variables.

Weighted Mode-Based Estimator: This method will deliver a consistent causal estimate if the largest number of similar individual-instrument causal effect estimates originates from valid instrumental variables.

## Results

Our comprehensive Mendelian Randomization analysis probed the relationship between green tea intake and various subtypes of lung cancer (Table 2).

**Table 2:** Results of Mendelian randomization analysis.

**Outcome**	**Data source**	**Method**	***n* SNPs**	**OR**	**Lower limit of 95%CI**	**Upper limit of 95%CI**	***P*-value**
Non-small cell lung cancer, adenocarcinoma	FinnGen databases	MR Egger	20	0.995	0.94	1.06	0.890204
		Weighted median	20	0.992	0.95	1.03	0.746656
		Inverse variance weighted	20	1.007	0.97	1.05	0.692271
		Simple mode	20	0.987	0.92	1.06	0.734049
		Weighted mode	20	0.977	0.92	1.04	0.427744
Non-small cell lung cancer, adenocarcinoma	The IEU Open GWAS	MR Egger	12	0.991	0.94	1.05	0.791947
		Weighted median	12	0.985	0.96	1.02	0.34669
		Inverse variance weighted	12	0.98	0.95	1.02	0.233971
		Simple mode	12	1.01	0.95	1.06	0.776474
		Weighted mode	12	1.008	0.96	1.06	0.793913
Non-small cell lung cancer, squamous	FinnGen databases	MR Egger	20	1.06	0.98	1.15	0.154438
		Weighted median	20	1.04	0.98	1.1	0.24092
		Inverse variance weighted	20	1.02	0.98	1.06	0.322191
		Simple mode	20	0.994	0.92	1.07	0.898038
		Weighted mode	20	1.05	0.97	1.14	0.238938
Non-small cell lung cancer, squamous	The IEU Open GWAS	MR Egger	12	1.05	1.01	1.1	0.058696
		Weighted median	12	1.007	0.98	1.03	0.65404
		Inverse variance weighted	12	1.001	0.98	1.02	0.920803
		Simple mode	12	1.01	0.96	1.06	0.688296
		Weighted mode	12	1.009	0.96	1.05	0.698975
Small cell lung cancer	FinnGen databases	MR Egger	20	1.11	0.99	1.24	0.094093
		Weighted median	20	1.04	0.96	1.13	0.344022
		Inverse variance weighted	20	1.05	0.99	1.11	0.130286
		Simple mode	20	1.04	0.92	1.18	0.528023
		Weighted mode	20	1.04	0.94	1.15	0.458009

### Non-small cell lung cancer, adenocarcinoma

Initially, we evaluated the association between green tea consumption and non-small cell lung cancer, specifically adenocarcinoma, using two independent datasets from FinnGen databases and the IEU Open GWAS. Our primary IVW analysis consistently indicated a non-significant association for both datasets (OR = 1.007, 95% CI: 0.97 to 1.05, p = 0.69; OR = 0.981, 95% CI: 0.95 to 1.02, p = 0.23 respectively). This non-significant effect was corroborated by several alternative MR techniques including MR Egger, Weighted Median, Simple Mode, and Weighted Mode, reinforcing the robustness of our results.

### Non-small cell lung cancer, squamous

For squamous cell lung cancer, another subtype of non-small cell lung cancer, our primary IVW analysis revealed a similar pattern of non-significant associations across datasets from FinnGen databases and the IEU Open GWAS (OR = 1.022, 95% CI: 0.98–1.06, *p* = 0.32; OR = 1.001, 95% CI: 0.98–1.02, *p* = 0.92 respectively). The robustness of these findings was reinforced by additional MR methods, suggesting a lack of a strong causal effect of green tea intake on the risk of squamous cell lung cancer.

### Small cell lung cancer

Regarding small cell lung cancer, our IVW analysis did not indicate a significant association between green tea intake and disease risk (OR = 1.048, 95% CI: 0.99–1.11, *p* = 0.13). Additional MR methods, including MR Egger, Weighted Median, Simple Mode, and Weighted Mode, echoed these non-significant findings.

Collectively, our comprehensive Mendelian Randomization analysis, encompassing multiple lung cancer subtypes and employing a suite of MR methodologies, consistently suggested a lack of significant causal relationships between green tea intake and lung cancer risk. However, the complexity of cancer etiology and potential pleiotropic effects of genetic instruments warrant a cautious interpretation of our findings.

## Discussion

In the current Mendelian randomization study, we sought to clarify the potential causal relationship between green tea consumption and lung cancer risk, which, to our knowledge, represents the first attempt to explore this relationship from a genetic epidemiology standpoint. We systematically examined different subtypes of lung cancer, including non-small cell lung cancer with adenocarcinoma and squamous cell carcinoma subtypes, and small cell lung cancer. Through both primary inverse-variance weighted analyses and various sensitivity analyses, our results consistently showed no significant associations between green tea intake and lung cancer risk.

Our conclusions stand in stark contrast to a considerable volume of prior research, which has observed a potential protective effect of green tea consumption against lung cancer, particularly among non-smokers (14, 16). This finding aligns with Wang et al.'s meta-analysis of case-control and cohort studies (23). In this meticulous assessment of daily tea consumers vs. infrequent tea drinkers, a statistically significant decreased risk of lung cancer was noted among daily consumers, suggesting a dose-response relationship. The observed protective effect was particularly striking in Asian populations, potentially indicating genetic or lifestyle interactions.

Despite these persuasive findings emerging from observational studies and the synthesis of multiple individual studies in meta-analyses, our Mendelian randomization analysis does not corroborate these protective associations. This dichotomy underscores the complexity of diet-cancer relationships and the methodological challenges in their robust examination (24, 25). It highlights the importance of diverse research methodologies, such as Mendelian randomization, in producing comprehensive and reliable insights, which might sometimes run counter to the prevailing narrative based on observational studies.

The discrepancy can be attributed to the inherent limitations of observational studies, which are subject to confounding bias and reverse causation. For instance, individuals who regularly consume green tea might adopt other healthier lifestyle habits such as regular exercise, balanced diet, and abstaining from smoking, which independently reduce lung cancer risk. Conversely, Mendelian randomization analysis leverages genetic variants as instrumental variables, which are not influenced by these lifestyle factors, thereby providing more robust and unbiased causal effect estimates.

While our Mendelian randomization analysis did not reveal a significant protective effect of green tea intake against lung cancer, we believe it is essential to highlight the substantial body of research that underscores the potential health benefits of green tea. Notably, green tea is enriched with polyphenols, particularly a catechin known as epigallocatechin-3-gallate (EGCG), recognized for its antioxidant, anti-inflammatory, and anticarcinogenic properties (1–4, 26). These properties have been substantiated by numerous *in vitro* and *in vivo* studies (27–29), which offer compelling evidence of the potential anti-cancer attributes of green tea and EGCG. These bioactive compounds are implicated in several crucial anticancer mechanisms, such as arresting the cell cycle, inducing apoptosis in cancer cells, and inhibiting angiogenesis, processes that could stymie the progression and proliferation of cancer cells. While our study does not confirm the protective effect of green tea consumption on lung cancer at a population level, the biological plausibility for green tea's anticancer potential cannot be entirely dismissed.

The strengths of our study are multifaceted. We adopted a stringent selection process for the genetic instruments for green tea intake, ensuring they were strongly associated with the exposure and were not linked to known confounders or in linkage disequilibrium. By employing several MR methodologies, each providing different assumptions and robustness to pleiotropy, we were able to provide a comprehensive and rigorous assessment of the causal effect.

Despite the novel insights offered by our study, we must recognize and address several potential limitations. Primarily, our reliance on self-reported green tea consumption data might be subjected to reporting bias. Participants could inadvertently overstate or understate their green tea consumption, which could introduce errors into our analysis. Implementing more objective and standardized measures of green tea intake, such as biomarkers, might help to attenuate this potential bias in future research.

Further, we must consider the inherent variability in green tea consumption across different populations. Variations in tea brewing methods, types of green tea consumed, and the specific green tea components consumed could introduce heterogeneity into our exposure measurement. For instance, brewing temperature and duration, as well as the part of the tea plant used, can significantly influence the concentration of polyphenols and other bioactive compounds in the tea (30, 31). Future studies might benefit from capturing this variability more accurately, possibly by incorporating data on specific tea brewing and consumption practices.

Another potential limitation pertains to the demographic specificity of our study cohort. Our analysis was rooted in data from populations of European descent, which could limit the generalizability of our findings. Genetic differences, dietary habits (32), and lifestyle factors (33) vary widely across different ethnic and geographic groups (34), and these factors might interact with green tea consumption in influencing lung cancer risk. Consequently, caution should be exercised in extrapolating our findings to other populations. Future research could aim to replicate our findings in diverse ethnic and demographic groups, thereby enhancing the global applicability and robustness of our conclusions.

Taken together, while our study provides valuable insights into the relationship between green tea consumption and lung cancer risk, the above limitations underscore the need for continued exploration in this area.

## Conclusion

In this Mendelian Randomization study, we rigorously examined the causal link between green tea consumption and various lung cancer subtypes, employing robust statistical methodologies. Our analyses, across multiple sensitivity tests, consistently show no significant causal effect of green tea intake on lung cancer risk, including adenocarcinoma, squamous cell, and small cell lung cancers.

Though our results indicate a null relationship with lung cancer risk, they should not negate the potential health benefits of bioactive compounds in green tea, such as EGCG. The complex etiology of lung cancer and the potential pleiotropic effects of our genetic instruments warrant cautious interpretation. For future studies, we recommend larger and more diverse cohorts, as well as improved instrumental variables to address confounding factors. Such investigations are vital for a nuanced understanding of green tea's role in lung cancer etiology.

## Data availability statement

Publicly available datasets were analyzed in this study. This data can be found here: https://gwas.mrcieu.ac.uk/, https://www.finngen.fi/en.

## Author contributions

JL: Data curation, Formal analysis, Methodology, Supervision, Validation, Writing – original draft, Writing – review & editing. YL: Data curation, Methodology, Validation, Writing – original draft. JJ: Data curation, Methodology, Validation, Writing – original draft. LG: Resources, Writing – review & editing. ZS: Validation, Writing – review & editing. CY: Data curation, Writing – review & editing. PL: Data curation, Writing – review & editing. MK: Conceptualization, Project administration, Resources, Supervision, Validation, Writing – review & editing.
